# Primary school teachers’ opinions and attitudes towards stuttering in two South African urban education districts

**DOI:** 10.4102/sajcd.v63i1.157

**Published:** 2016-07-27

**Authors:** Kristen Abrahams, Michal Harty, Kenneth O. St. Louis, Lehana Thabane, Harsha Kathard

**Affiliations:** 1 Department of Communication Sciences and Disorders, University of Cape Town, South Africa; 2 Department of Communication Sciences and Disorders, West Virginia University, United States; 3 Department of Clinical Epidemiology and Biostatistics, McMaster University, Canada; 4 Department of Health Sciences Education, University of Cape Town, South Africa

## Abstract

**Background:**

As teachers form an important part of the intervention process with children who stutter in primary school, the primary aim was to describe primary school teachers’ attitudes in South Africa. The secondary aim was to compare teachers’ attitudes towards stuttering in South Africa with those from a pooled group of respondents in the Public Opinion Survey of Human Attributes–Stuttering (POSHA-S) database from different countries collected in 2009–2014.

**Method:**

A quantitative, cross-sectional survey research design was used. Primary schools in two education districts in Western Cape, South Africa, were sampled. The POSHA-S, a self-administered questionnaire, was completed by a cluster sample of 469 participants.

**Results:**

Overall positive attitudes towards stuttering were found, specifically related to the potential of people who stutter, although the result should be interpreted with caution as the sample was not homogenously positive. Teachers still had misconceptions about personality stereotypes and the cause of stuttering. The attitudes of the South African sample were slightly more positive compared with the samples in the current POSHA-S database.

**Conclusion:**

When developing stuttering intervention strategies, there are a number of key considerations to take into account. The study provides a basis for speech-language therapists to think about intervention with teachers and which areas of stuttering to consider.

## Introduction

It is well-documented in the literature that people in the child’s environment influence the child’s experiences of their stutter (Bennett, 2003; Blood & Blood, 2004), communicative ability and their progress in therapy (Murphy, Yaruss & Quesal, 2007). For school-aged children who spend a large amount of time at school, there can be little dispute that teachers are figures of authority who can have a significant impact on a child’s early years. If teachers hold unsubstantiated views on stuttering, it can have a negative impact on how they perceive and interact with children who stutter (CWS; Abdalla & St. Louis, 2012). Additionally, teachers’ empathy and behaviour as they interact with CWS can have an impact on the way these children are viewed and treated by their peers (Boberg & Calder, 2012; Jenkins, 2010).

The success of the therapeutic process is highly dependent on understanding the attitudes of significant others (including teachers) in CWS’ lives (Abdalla & St. Louis, 2012). Currently, little is known about teachers’ attitudes towards stuttering in South Africa. In the management of stuttering, teachers are a central component; therefore, understanding their attitudes will assist in planning and developing appropriate interventions. With limited knowledge and resources for the South African context, this leaves speech-language therapists (SLTs) with many questions:

- What do teachers know about stuttering?
- How do they feel about stuttering?
- How do they react?
- How do teachers manage stuttering in the classroom?

The present study aims to address these questions in the South African context.

International studies have found varying teacher opinions and attitudes towards stuttering. Pachigar, Stansfield and Goldbart (2011) conducted a mixed-method (i.e. quantitative and qualitative) study in Mumbai considering 58 Indian teachers’ attitudes towards CWS. The authors found that many teachers had not been provided with any formal information about stuttering and they reported having limited experience with CWS. The questionnaire found broadly positive attitudes towards CWS. The interview process also highlighted a positive approach to dealing with CWS in the classroom, specifically related to decreasing the pressure placed on the child, and subsequently reducing stress and anxiety. Similarly, Irani, Abdalla and Gabel (2012) aimed to determine Kuwaiti Arab teachers’ attitudes towards people who stutter (PWS) and to compare their attitudes with American teachers to determine if cultural differences were present. The results indicated that Arab teachers generally showed neutral to positive attitudes towards PWS. One-third of Arab teachers indicated negative attitudes on questions related to employment and social skills. Although both sets of teachers generally had positive attitudes towards stuttering, American teachers were significantly more positive than Arab teachers (*p* ≤ 0.003).

Due to the different questionnaires used in each study, drawing comparisons between attitudes is difficult. In order to address the lack of a standardised measure, the International Project on Attitudes Toward Human Attributes initiative developed the Public Opinion Survey of Human Attributes–Stuttering (POSHA–S) which is designed to measure public opinion on, and attitudes towards, stuttering worldwide (St. Louis, 2011). The POSHA–S considers attitudes in relation to other positive (i.e. intelligence), neutral (i.e. left-handedness) and negative (i.e. obesity, mental illness) human attributes. These attributes were included in the POSHA–S as ‘attitudes towards stuttering will be more meaningful within the context of attitudes towards other human conditions’ (St. Louis & Roberts, 2010, p. 362). To assess public opinion, the POSHA–S groups items together to form components and groups components to form subscores (St. Louis, 2012). Items related to stuttering are grouped according to two subscores: (1) Beliefs about Stuttering and (2) Self-reactions to Stuttering. Beliefs about Stuttering relate to a participant’s thoughts and impressions about stuttering (not related to them personally), whereas Self-reactions to Stuttering consider a participant’s self-perceptions of his or her behaviours, reactions and knowledge (Przepiorka, Blachnio, St. Louis & Wozniak, 2013).

Three recent POSHA–S studies (Abdalla & St. Louis, 2012; Arnold, Li & Goltl, 2015; Li & Arnold, 2015) investigated school teachers’ attitudes towards stuttering. Abdalla and St. Louis (2012) examined school teachers’ knowledge, attitudes and beliefs about stuttering. Participants were 262 Arabic-speaking residents of Kuwait who were in-service public school teachers of grades 1–12 and 209 pre-service school teachers, that is, education students. The results of the study found that teachers were familiar with stuttering, but held misconceptions about the cause of stuttering, personality stereotypes, role entrapment (i.e. cannot do any job they want) and strategies for coping with stuttering (i.e. repetition of word until the child is able to say it, filling in words, etc.; Abdalla & St. Louis, 2012). Abdalla and St. Louis (2014) further found that the implementation of an education documentary video depicting factual and emotional aspects of stuttering was found to positively change pre-service teachers’ attitudes (in terms of the subscores, Beliefs about Stuttering and Self-reactions to Stuttering) but not those of the in-service teachers.

More recently, Arnold *et al*. (2015) used the POSHA–S Beliefs subscore to compare 269 kindergarten to grade 12 teachers with 1388 non-teachers from the POSHA–S database. Linear regression analysis indicated that there were no differences between teachers and non-teachers. Nevertheless, irrespective of profession, female participants provided statistically significant (*p* < 0.001), more accurate beliefs. An increase in age (*p* < 0.01) and years of education (*p* < 0.01) were significantly associated with more favourable beliefs. Participants who knew at least one person who stuttered were found to have more positive responses (*p* < 0.0001). Li and Arnold (2015) further considered the Self-reactions subscore. The results indicated no significant difference in any of the components forming the self-reactions subscore except knowledge source. Teachers’ generally scored higher than the non-teachers, indicating a larger variety of sources of knowledge. Age (*p* < 0.001) and years of experience (*p* < 0.001) were also associated with more positive responses. Regardless of occupation, it was found that female participants generally scored higher than male participants for the accommodating and/or helping component (*p* < 0.001).

From the existing POSHA–S studies, it is clear that there are differences in opinions about stuttering worldwide, possibly because of the unique context of each country with regard to religion, culture, language, nationality and ethnicity (Abdalla & St. Louis, 2012; St. Louis, 2005). This highlights the importance of gaining a contextual understanding of stuttering internationally, especially in areas where little is known about the condition. South Africa provides a unique context for the provision of health care services. Apartheid has greatly shaped South Africa, creating social, economic and racial inequalities (Engelbrecht, Oswald & Forlin, 2006). The diversity within the country both linguistically and culturally adds to the complexity of the context.

In post-apartheid South Africa, policy changes in education led to desegregation of schools and many other changes to the education system and institutions. The teaching profession has had to cope with the movement to a single national system, as well as a change of curriculum, which acknowledges the importance of professional autonomy. Teachers were required to gain new knowledge and competencies with drastic changes to the composition of classrooms, demographically, linguistically and culturally (Department of Education, 2006).

In mainstream primary schools, teachers have little or no direct support from SLTs as the majority of SLTs employed by the Department of Education are based at a district level or in ‘special schools’ (Kathard *et al*., 2011). As a result, SLTs only serve a small minority of learners’, generally working with more severe cases (Kathard *et al*., 2011). Along with the well-documented language and literacy difficulties plaguing the education sector (Navsaria, Pascoe & Kathard, 2011), it is unlikely that a condition such as stuttering will be a priority.

Although international studies provide SLTs with insight into teachers’ attitudes, no study in South Africa has considered teachers and stuttering. Therefore, our present knowledge base on teachers’ attitudes towards stuttering is strongly influenced by the developed world. According to Kathard and Pillay (2013), the developed world’s knowledge base has been labelled as universal, when in fact it has been influenced by culture. Therefore, we cannot assume that the developed world’s knowledge will be identical to the knowledge of the developing world. For the South African context, which is so diverse, it is essential that we determine which aspects are universal and which are specific to cultural groups (Abdalla & St. Louis, 2012).

The study forms part of a larger ongoing project considering stuttering in the school context. This study is the first in the Western Cape to consider teachers’ attitudes; therefore, using the survey, we aimed to gain general insight into teachers’ opinions. As such, the study focused on urban areas in Cape Town, where the population density is higher. Further studies consider a more in-depth understanding through qualitative data. This study presents the findings of the survey considering teachers’ views on stuttering in the Western Cape in a context where teachers have reported concerns about stuttering (Kathard *et al*., 2014) alongside general communication challenges in the classroom context (i.e. linguistic diversity, lack of development of communication skills). The study also compared South African teachers’ attitudes to the POSHA–S database, in order to identify patterns of relative strengths and challenges for this group of teachers.

## Research method and design

A quantitative, cross-sectional survey design was used in the study. Before the administration of the POSHA-S, a pilot study was conducted to determine the adequacy of the research procedure, the potential response rate, the level of awareness of stuttering and the reliability and validity of the POSHA–S for the South African context. Based on the results of the pilot study, modifications were made to the phrasing of questions and the procedure.

### Participants

The present study was conducted in the Western Cape of South Africa, specifically two urban education districts, Metro East and Metro Central within Cape Town. Cluster sampling was used (Bruce, Pope & Stanistreet, 2008) to obtain a sample of teachers working in primary schools in the two education districts. In order to obtain a representative sample, schools were further stratified. Schools were stratified according to teaching phase (i.e. foundation [grades 1–3] or intermediate phase [grades 4–7]). Schools were also stratified according to quintile [i.e. government funding provided to schools based on resources and poverty scores (Motala, 2006; Sayed & Motala, 2012), i.e. higher (quintile 4 and 5) and lower (quintile 1 and 2)]. It should be noted that this study does not focus on the effect of these variables.

**FIGURE 1:**
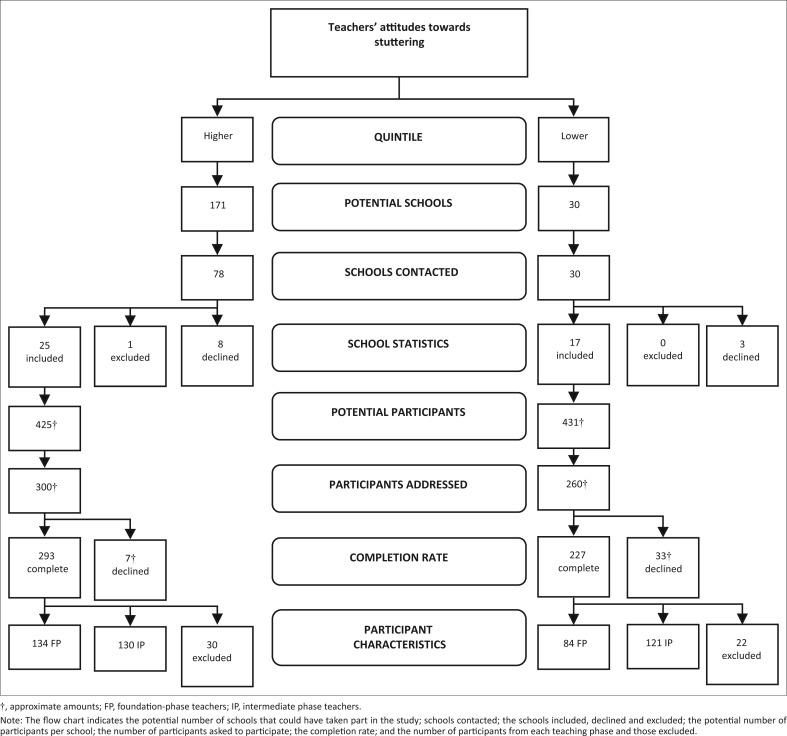
Flow chart depicting sampling process, with number of respondents per stage.

In 42 chosen schools, an approximate total of 560 out of 856 teachers (according to the school principals) were invited to participate. Figure 1 provides an overview of the schools and teachers sampled. Based on the figure, it is clear that there were a number of non-respondents, either because of declining to take part or logistical issues.

Five hundred and twenty participants agreed to complete the questionnaire with a response rate of ± 92.9% (i.e. 520/560). Fifty-two participants were excluded because of incorrect profession or critical information that was not completed. Table 1 summarises the demographic information for all participants’ in the sample. There were a total of 469 participants, with a mean age of 45 years with a range of 22–66.6 years.

**TABLE 1:** Demographic information for the South African participants of the study (*N* = 469).

Variable	Statistics	

	*n*	%
**Gender:**		
Male	98	20.8
Female	352	75.1
No response	19	4.1
**First language:**		
Afrikaans	113	24.1
English	128	27.3
IsiXhosa	181	38.6
Other	47	10
**Multilingual:**		
Second language	261	55.6
Third language	187	39.9

Mean age in years is 22.0–66.6.

*n* = 45.

### The Public Opinion Survey of Human Attributes-Stuttering

The POSHA–S was developed to be translatable into a variety of languages and cultures, efficient, valid, reliable and easy to use (St. Louis, 2005). South Africa also participated in development of POSHA–S (St. Louis, 2005). The POSHA–Experimental (i.e. previous version) was able to identify slight differences within sample groups such as distinctions between rural versus urban, student versus non-student and low- and/or middle-income versus high-income nations (St. Louis, 2005). Research has also found that the POSHA–S has a satisfactory test–retest reliability (St. Louis, Lubker, Yaruss & Aliveto, 2009), internal consistency (Al-Khaledi, Lincoln, McCabe, Packman & Alshatti, 2009) and construct and concurrent validity (St. Louis *et al*., 2009). For a description of the layout of the POSHA–S, refer to Ip, St. Louis, Myers and Xue (2012).

To assess public opinion, the POSHA–S groups items together to form components and groups components to form subscores (St. Louis, 2012). The mean scores for test items formed the component scores, and the mean for certain component scores formed the three subscores. The mean of the two stuttering subscores formed the Overall Stuttering Score. All the rating scores were then converted using a scale from −100 to +100 with neutral = 0 (St. Louis, 2012). Depending on the wording of the questions, the sign (i.e. + or −) was reversed so that the higher the overall score, the more favourable the attitude (St. Louis, 2012).

For the present study, modifications were made (i.e. changing wording, adding items to questions and adding additional demographic questions) to the POSHA–S in order to enhance the content validity of the study to better suit the South African context. Questions related to teachers’ views on management of stuttering in the classroom were included and were adapted from Crowe and Walton (1981), Yeakle and Cooper (1986) and Heite (2000).

Comparisons can be drawn to the median, lowest and highest sample means in the POSHA–S database so that researchers are able to determine the extent to which their sample is comparable or different from previous research samples (St. Louis, 2011). Circa August 2014, the database consisted of 10 174 participants representing 36 countries and 22 languages. Samples were gathered from many different professions including learners, family members of a PWS, and even food and hospitality service workers (St. Louis, 2015). The highest Overall Stuttering Score to date was obtained from a sample of stuttering, self-help leaders, suggesting that they held the most favourable attitudes towards stuttering, with the lowest score from a group of mid-socioeconomic status parents in Karnataka, India, suggesting they held the least favourable (St. Louis, 2015).

### Procedure

Informed consent was carried out according to guidelines of the Health Science Research Ethics Committee and the Western Cape Department of Education. Potential participants were identified by principals of the selected schools who verbally consented to the study, where upon information letters were distributed to the school for all potential participants. After agreeing on times, and locations, the teachers met in a group setting where consent was obtained and questionnaires were handed out for immediate completion. The participants were asked to complete the questionnaires individually.

### Data analysis

The distribution of the sample was determined using frequency tables. Percentages and bar charts were used to give an overview of the responses of the participants for each section in order to describe the views of all primary school teachers sampled.

The data from this study were then compared to the average calculated from all the data from the POSHA–S database (St. Louis, 2012), in order to determine similarities and differences between responses obtained in the present study and previous study samples (St. Louis, 2011). In addition, percentile ranks of the mean scores for the items, components, subscores and Overall Stuttering Score were compared to samples in the database. The quartile in which the results fell in were also determined (i.e. first quartile [0%–25%]; interquartile [25%–75%] and fourth quartile [75%–100%]), in order to determine whether South African teachers’ attitudes were more or less positive than the other respondents in the database (Özdemir, St. Louis & Topbas, 2011). A radial graph was used to show the mean ratings for the components and subscores from all of the samples in the POSHA–S database and the lowest, median and highest mean ratings (Przepiorka *et al*., 2013).

## Ethical considerations

The following ethical principles were considered and incorporated into the study (World Medical Association Declaration of Helsinki, 2013): autonomy, beneficence and non-maleficence and justice.

## Results

The overall key results are presented according to the stuttering subscores, Beliefs about Stuttering and Self-reactions to Stuttering. In general, positive results were found, particularly related to PWS’s potential and their reactions to stuttering.

### Beliefs about stuttering

Over 50% of participants indicated that PWS were nervous or excitable (56%, *n* = 248) or shy or fearful (58.2%, *n* = 256). However, over 80% of participants did not agree with the statement that PWS have themselves to blame for their stutter (84.1%, *n* = 376).

Participants’ beliefs about the cause of stuttering were diverse. While majority of participants (53.4%, *n* = 230) indicated that stuttering was caused by genetic inheritance, others believed that it was caused by an act of God (36.3%, *n* = 153), learning or habits (26.7%, *n* = 113) or a very frightening event (24.2%, *n* = 103).

Participants generally believed that a SLT should assist PWS (94.1%, *n* = 416). Other participants were divided on their views with 38.7% (*n* = 163) indicating that people like themselves should assist PWS while 43.5% (*n* = 183) disagreed. Over 50% of participants indicated that PWS should not be helped by a medical doctor or other PWS, with 93.8% (*n* = 390) indicating that traditional healer would not be an appropriate referral.

### Self-reactions to stuttering

The majority of participants indicated that they would do the following: try to act like the person was talking normally (81.5%, *n* = 347), feel comfortable and relaxed (79.5%, *n* = 348) and speak calmly and slowly to the person (67.4%, *n* = 295).

Many participants indicated that they personally knew someone who stutters (83.7%, *n* = 379), with 25.6% (*n* = 116) who currently had someone in their class who stutters. Twenty-one (4.7%) participants indicated that they stuttered. While the majority of participants had experience with stuttering, participants’ indicated that they knew the least about stuttering (*M* = 2.5) and mental illness (*M* = 2.6) in comparison to the other human attributes (i.e. intelligence [*M* = 3.6], left-handedness [*M* = 3.2] and obesity [*M* = 3]).

### Comparison to Public Opinion Survey of Human Attributes-Stuttering Database

**FIGURE 2:**
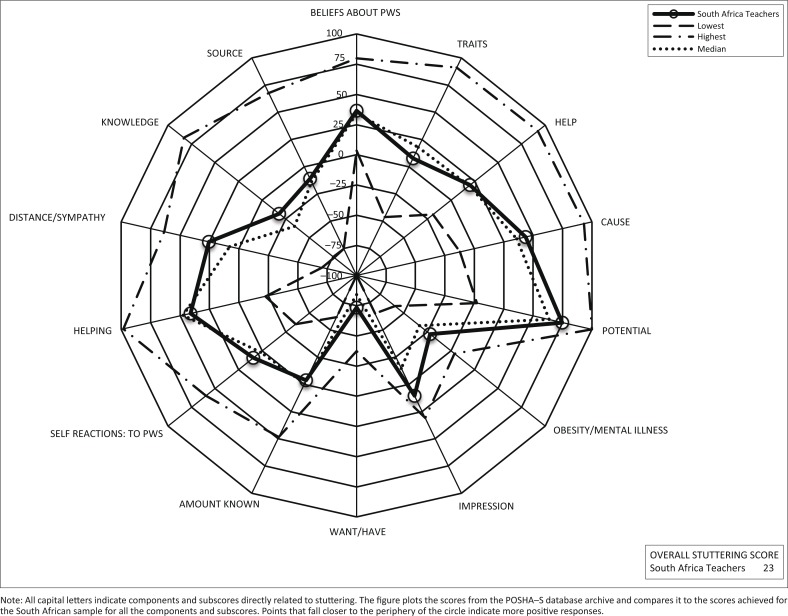
Summary of POSHA–S radial graph for the South African sample in comparison to the lowest, median and highest scores in the POSHA–S database archive (circa August 2014).

The data from the present study are compared with the lowest, highest and median samples from the POSHA-S database to date (circa August 2014). Figure 2 is a visual representation of how the responses of the South African sample compares to the database in terms of the components, subscores and overall score on the POSHA-S.

In order to provide a percentile comparison, mean scores for all of the POSHA–S ratings (items, components, subscores and Overall Stuttering Score) for the South African sample were compared to the 146 samples in the POSHA–S database (refer to Table 2).

**TABLE 2:** South African participants’ mean ratings for the Overall Stuttering Score, subscores and components followed by percentile values.

POSHA–S variables	South African participants	South African participants in %	Median score POSHA–S database
**Beliefs about PWS:**	**37**	**54**	**34**
Traits and/or personality	8	38	18
Help	20	52	19
Cause of stuttering	44	65	36
Potential	75	81	64
**Self-reactions to PWS:**	**9**	**73**	**2**
Accommodating and/or helping	41	33	47
Social distance and/or sympathy	25	73	8
Knowledge and/or experience	-18	84	-34
Knowledge source	-11	53	-14
**Obesity and/or mental illness:**	**-22**	**91**	**-34**
Overall impression	10	94	-15
Want to be	-73	85	-84
Amount known about	-4	41	-1
**Overall stuttering score**	**23**	**62**	**18**

Note: Also shown are median scores from the Public Opinion Survey of Human Attributes–Stuttering (POSHA–S) database.

The South African sample scored higher for all subscores and the Overall Stuttering Score (refer to Table 2). The South African sample scored lower than average for (1) Traits and/or Personality, (2) Accommodating and/or Helping (3) and the Amount Known About Obesity and Mental Illness. Also within the cause component, specifically the response act of God was much lower than the POSHA–S database (15 for the South African sample compared to 65 for the POSHA–S database; not shown in Table 2). Within the Traits and/or Personality component, the items which received the lowest score were PWS are nervous or excitable (a mean score of -26 for the South African sample compared to three for the database) or shy and fearful (a mean score of -28 vs. -8). Within the Accommodating and/or Helping component, the greatest difference between scores was found for the item, if I were talking with a person who stutters, I would feel comfortable or relaxed (South African sample scored -22 compared to 22 for the database).

Two stuttering components fell into the fourth quartile, namely Potential and Knowledge and/or Experience. For Potential, the items that received the highest score were *PWS* can make friends (score of 91 compared to 93), lead normal lives (94 compared to 87) and can do any job they want (78 compared to 51). For the Knowledge and/or Experience component, personal experience with stuttering received the highest score (49 for South African sample compared to four for the database).

For all 60 standard POSHA–S ratings, (i.e. all items, components, subscores and Overall Stuttering Score), 5% of the South African means were in the lowest quartile, 62% were in the interquartile range and 33% were in the highest quartile. As the majority of the scores fell within the interquartile range, it can be assumed that the majority of the attitudes of the South African participants in this study were generally aligned with the typical results found in other studies. Nevertheless, a considerable percentage of scores were in the highest quartile and few in the lowest quartile, indicating that the South African sample had relatively more positive attitudes than average. Supporting this result is the fact that the overall score for the South African sample (i.e. 23) was slightly higher than the median sample mean of 18.

## Discussion

It is encouraging that there was a general consensus from primary school teachers that regardless of a person’s stutter, teachers believed that PWS had the potential to lead successful lives both socially and economically. Teachers were generally familiar with stuttering and would act in a considerate manner when interacting with a CWS.

However, personality stereotypes were still evident (i.e. PWS are shy and/or fearful or nervous and/or excitable). There was also a large diversity of responses for the cause of stuttering ranging from genetic inheritance to an act of God.

Due to the limited amount of SLTs in the schooling system, the responsibility for managing stuttering in the classroom largely falls upon the teacher. With primary school teachers reporting a lack of perceived knowledge about stuttering as compared to the other human attributes (i.e. intelligence, left-handedness, mental illness and obesity), there are a number of key findings that can assist in conceptualising and developing interventions targeted at teachers.

Some teachers (43.5%) did not believe they should be someone who helps PWS. This result may also be linked to the fact that teachers do not feel they have the knowledge and skills necessary to adequately assist a CWS (Berquez, Cook, Millard & Jarvis, 2011). We agree with others that teachers are key figures in the lives of CWS as they are one of the communication partners for children in the classroom (Gottwald & Hall, 2003). Therefore, it is important that SLTs are able to empower teachers with the basic knowledge and skills to manage CWS in the classroom. With a general consensus that improved knowledge can lead to improved attitudes (Crowe & Walton, 1981; Hobbs, 2012; Yeakle & Cooper, 1986), implementation of professional development programmes related to stuttering may therefore improve teachers’ knowledge about stuttering and subsequently their beliefs. Also, as a consequence of increased knowledge, teachers may feel more confident in their ability to assist a CWS in the classroom. The study by Abdalla and St. Louis (2014) found that the implementation of an education documentary video positively changed certain aspects of student teachers’ attitudes towards stuttering. The use of such educational material could prove to be a valuable asset when attempting to change attitudes of individuals who hold negative or unfavourable attitudes towards PWS. Their results provide insight into the potential strategies that could be employed in order to address the unfavourable and reinforce positive attitudes. Kathard *et al*. (2014) found that through collaboration with teachers as intervention partners, negative attitudes peers hold towards CWS may be reduced. Educating teachers about stuttering and how to manage stuttering in the classroom were essential to the success of the study. This leads to the question: *What kind of information do teachers require?*

Participants in this study generally believed that PWS could be productive members of society. Even with a perceived lack of knowledge about stuttering, it did not affect their opinion about the potential of PWS. This result is encouraging for intervention planning because a positive attitude towards the potential of PWS is essential for the success of any intervention programme (Snyder, 2001).

Results generally indicated that teachers would be considerate when interacting with a PWS. Many teachers would try to act like the person was talking normally (81.5%) and speak calmly and slowly to the person (67.4%). Teachers also indicated that they would not feel impatient (89.2%) or pity (64.9%). It has been made clear that the environmental context in which a child communicates is important relative to the reactions of teachers and peers, which can affect the child’s fluency (Yaruss & Reardon, 2002). Conture *et al*. (2006) highlight the importance of ‘pressure-free’ response time for CWS within the educational setting. Accordingly, the results from our study are encouraging as teachers’ report acting in a way which seems to promote fluency through creating an environment which does not put too much pressure on the CWS.

The results also highlight some differences in the beliefs and attitudes of South African teachers compared to the individuals in the POSHA–S database. There were two stuttering scores that were lower than the POSHA–S database, namely: Traits and/or Personality component and the item act of God as a cause of stuttering. For the former, the items which contributed to the lower score for the South African sample related to personality stereotypes, that is, shy or fearful (28th percentile) or nervous or excitable (20th percentile). MacKinnon, Hall and Macintyre (2007) theorised that stuttering stereotypes originated from the anchor-adjustment hypothesis, which states that ‘people adopt another person’s perspective by serially adjusting from their own perspective’ (p. 300). Their study concluded that the formation of the stuttering stereotype was because of generalisations and adjustments that fluent speakers make, based on their own personal experience of normal dysfluency. Teachers may, therefore, believe that PWS possess certain personality stereotypes based on their own negative experiences with normal dysfluency. Coupled with the fact that the present study found that teachers perceived they had limited knowledge on stuttering and that they generally obtained this knowledge from personal experience, the anchor-adjustment hypothesis may be a viable explanation.

In many African cultures, disability and illness are viewed within a spiritual framework (Legg & Penn, 2013); thus, the belief that stuttering is caused by an act of God (22nd percentile) was expected to be more prevalent than in North America or Europe. The result clearly shows that populations may vary in how they view potential causes of stuttering. As a consequence, interventions may be required to be specifically tailored to the targeted community. The results also highlight that SLTs cannot assume that all communities will respond to stuttering in the same way and this reinforces the importance of gaining a nuanced understanding of the views of the community before the implementation of any professional development programme. The study provided a first-level survey to inform intervention planning. Presently, there are further studies exploring teachers’ attitudes in greater depth (Hobbs *et al*., 2016).

Compared to the findings of Abdalla and St. Louis (2012), South African teachers provided more positive responses relating to the potential of PWS and their reactions towards PWS. Both groups of participants had misconceptions about the cause of stuttering and who should help a PWS. However, misconceptions differed, for example, many Arab teachers believed stuttering was caused by a very frightening event compared to few South African teachers. Similarly, half of the Arab teachers thought that a medical doctor should help a PWS compared to very few South African teachers. The comparison highlights the importance of gaining knowledge about teachers’ attitudes within the context in which they work as clearly South African and Arab teachers, while similar in some regards, had differing opinions and thoughts about stuttering. The studies by Li and Arnold (2015) and Arnold *et al*. (2015) further highlight that there are a number of variables, including gender, age, years of experience and familiarity that have an impact on attitudes.

Teachers’ belief that PWS have the potential to be productive members of society provides a positive platform for intervention. Intervention should reinforce positive attitudes and/or behaviours such as the considerate classroom management strategies and reactions to stuttering and focus on addressing misconceptions related to personality stereotypes and the causes of stuttering in order to improve teachers’ knowledge and subsequently their belief that they too can help PWS.

While the overall result indicates a positive attitude of the sample, a closer examination of the findings indicated that the majority of responses for the South African sample fell within the interquartile range (62%) when compared to the database archive. As a result, the findings of the study should be interpreted with caution. The overall result may mask the fact that there is still a substantial percentage of teachers who hold negative attitudes towards stuttering. The sample is not homogeneous, as highlighted by the significant differences reported on certain demographic factors investigated. Furthermore, participants who respond to questionnaires are generally more positively inclined (Jenkins *et al*., 2013). Teachers who were more positive towards stuttering are more likely to participate in the study than teachers who were not. In all questionnaires, there is a possibility that the participants might provide responses that are more socially acceptable than their true beliefs (i.e. the Hawthorne effect; Benson, 2004). Teachers may have thought that negative attitudes towards PWS may be viewed as unacceptable for their profession (Irani & Gabel, 2008). Individuals may be reluctant to overtly state negative attitudes because of the predisposition of society to political correctness (Irani & Gabel, 2008). It is also important to take into consideration that the non-respondents (those who did not complete the questionnaire) may have held more negative views towards stuttering. As a result, caution should be exercised when generalising the findings of the study to the broader teaching profession.

## Strengths, limitations and future research

As this is the only known study to consider South African teachers’ attitudes towards stuttering, it provides a platform for understanding attitudes within the country’s primary school system in the urban area of the Western Cape and provides clinicians with insight into intervention planning. The large sample size makes the study robust relative to the generalisability of results. However, given the diversity of schools in South Africa, the applicability of findings to other geographical areas must be made with caution.

The following limitations of the study are acknowledged and were kept in mind during the interpretation of the results:

- The results for all participants who agreed to participate in the study were utilised in the analysis. On analysis of the results, it was found that there were participants who did not complete the majority of the questionnaire. In future, it is suggested that the quality of the responses be determined in order to decrease the possibility of inaccuracies in the analysis
- The validity and reliability of the added questions and options were not formally tested and established. However, as the primary aim was to describe teachers’ attitudes, determining the validity and reliability of the additional questions and options was not a main priority.

Future research may focus on:

- Qualitative analysis of teachers’ attitudes to gain a more in-depth understanding, not only of what attitudes and reactions teachers have towards stuttering but also the important element of why.
- The attitudes of teachers in rural areas. The present study focused on urban population and previous research (Doody, Kalinowski, Armson & Stuart 1993) has shown that attitudes between urban and rural communities may differ.
